# Hydration in Deep Eutectic Solvents Induces Non-monotonic Changes in the Conformation and Stability of Proteins

**DOI:** 10.1021/jacs.2c11190

**Published:** 2022-12-16

**Authors:** Adrian Sanchez-Fernandez, Medina Basic, Jenny Xiang, Sylvain Prevost, Andrew J. Jackson, Cedric Dicko

**Affiliations:** † Centro Singular de Investigación en Química Biolóxica e Materiais Moleculares (CIQUS), Universidade de Santiago de Compostela, Rúa de Jenaro de la Fuente, s/n, Santiago de Compostela 15705, Spain; ‡ Food Technology, Engineering and Nutrition, Lund University, Box 124, Lund 221 00, Sweden; § Institut Laue-Langevin, DS / LSS, 71 Avenue des Martyrs, Grenoble 38000, France; ∥ European Spallation Source, Box 176, Lund 221 00, Sweden; ⊥ Department of Physical Chemistry, Lund University, Box 124, Lund 221 00, Sweden; # Pure and Applied Biochemistry, Department of Chemistry, Lund University, Box 124, Lund SE-221 00, Sweden; ¶ Lund Institute of Advanced Neutron and X-ray Science, SE-223 70 Lund, Sweden

## Abstract

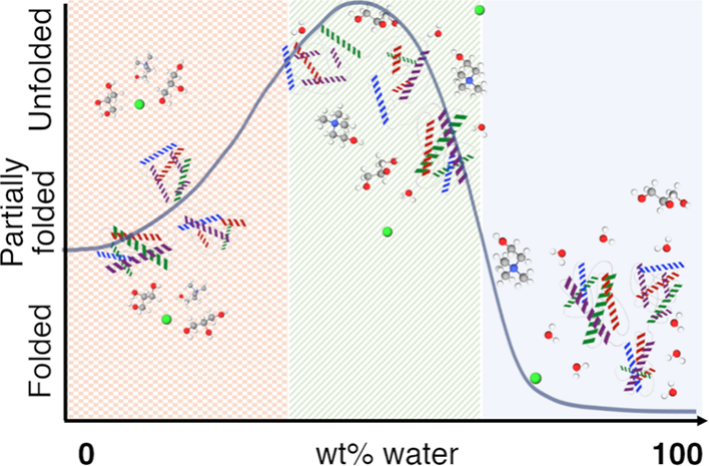

The preservation of labile biomolecules presents a major challenge in chemistry, and deep eutectic solvents (DESs) have emerged as suitable environments for this purpose. However, how the hydration of DESs impacts the behavior of proteins is often neglected. Here, we demonstrate that the amino acid environment and secondary structure of two proteins (bovine serum albumin and lysozyme) and an antibody (immunoglobulin G) in 1:2 choline chloride:glycerol and 1:2 choline chloride:urea follow a re-entrant behavior with solvent hydration. A dome-shaped transition is observed with a folded or partially folded structure at very low (<10 wt % H_2_O) and high (>40 wt % H_2_O) DES hydration, while protein unfolding increases between those regimes. Hydration also affects protein conformation and stability, as demonstrated for bovine serum albumin in hydrated 1:2 choline chloride:glycerol. In the neat DES, bovine serum albumin remains partially folded and unexpectedly undergoes unfolding and oligomerization at low water content. At intermediate hydration, the protein begins to refold and gradually retrieves the native monomer–dimer equilibrium. However, *ca.* 36 wt % H_2_O is required to recover the native folding fully. The half-denaturation temperature of the protein increases with decreasing hydration, but even the dilute DESs significantly enhance the thermal stability of bovine serum albumin. Also, protein unfolding can be reversed by rehydrating the sample to the high hydration regime, also recovering protein function. This correlation provides a new perspective to understanding protein behavior in hydrated DESs, where quantifying the DES hydration becomes imperative to identifying the folding and stability of proteins.

## Introduction

Conformation and dynamics are overarching concepts in understanding protein stability, enzymatic activity, and molecular recognition.^1−4^ The use of proteins in their natural aqueous environment is restricted to a limited variety of conditions as proteins are marginally stable outside their native conformational window.^5^ The development of non-aqueous enzymology extended the range of possible solvents for protein stabilization, potentially improving protein stability and substrate specificity.^6^ Notably, organic solvents also allowed tailoring protein behavior, such as the selectivity of an enzymatic route toward specific products, through changes in the solvent properties.^7^ More recently, the alternatives for non-aqueous protein stabilization and function were further expanded by using ionic liquids as solvation environments.^8^ Progress has been made in understanding protein behavior in these complex solvents, where subtle changes in the solvent characteristics can lead to a wide variety of biophysical and enzymatic responses.^9,10^

Deep eutectic solvents (DESs) are eutectic mixtures that show a larger depression in the melting point (“deep”) than that predicted for an ideal mixing of their constituents.^11^ In particular, the majority of DESs discovered heretofore belong to the *Type III* category. Those are formed by an organic salt (typically ammonium) and a neutral compound, acting as a hydrogen bond donor, at the eutectic ratio of the mixture. Mixing these precursors leads to a highly entropic state stabilized by an extensive hydrogen bond network, often remaining liquid at room temperature.^12−15^ DESs are compared to ionic liquids since different precursors allow great control over the solvent physicochemical properties, such as polarity, hydrophobicity, and hydrogen potential.^11,16−19^ Importantly, DESs can be synthesized from bio-derived compounds, such as sugars, carboxylic acids, and ammonium salts, which bestows the system with mild character, thermal and chemical stability, sustainability, and biocompatibility.^20−24^ Hence, DESs offer a tailorable, suitable environment for biomolecules, probing to be milder alternatives to ionic liquids.^8^ Recently, particular emphasis has been placed on developing DESs as solvents for biomolecule assembly, preparation of functional biomaterials, enzymatic catalysis, and preservation of biomolecule integrity, among others.^20,23,25−33^

Despite the promising outlook for biotechnological applications using DESs, the inherent properties of the solvent (e.g., high viscosity, low ion mobility) often hinder further potential developments. In this sense, proteins present a challenge for incorporation into DESs as their solubilization rate is extremely slow, and their thermo-labile character precludes incorporation through heating.^34−36^ An accepted route to leverage these properties of DESs is adding water to the system.^37,38^ This approach offers an extra degree of freedom for tailoring the solvent properties, for example, by significantly reducing the viscosity of the DESs and improving the dissolution rate of recalcitrant macromolecules.^38^ Consequently, most investigations on proteins in DESs have employed hydrated versions of these solvents. For instance, many studies have shown that the activity of different enzymes is retained in hydrated DESs.^26,36,39−42^ Also, the addition of sufficient water to DESs causes proteins to retrieve their native structure.^35,36,42−44^ However, lower levels of solvent hydration could influence the behavior of the protein as water can either modify or disrupt the molecular interactions within the DESs.^45−47^ This has been widely explored for hydrated organic solvents and ionic liquids, showing that adding water can induce nonlinear responses in protein behavior and denaturation.^7,48,49^ Also, it has been shown that DESs can promote the crystallization of proteins under certain hydration conditions, which control the nucleation rate.^50^ Surprisingly, the effect of hydration is often neglected when studying the solution behavior of proteins in hydrated DESs.

To fill this knowledge gap, we present an investigation of protein behavior in hydrated DESs across a wide range of hydration levels using UV–vis absorption spectroscopy and far-UV circular dichroism (CD). The solvation environment and secondary structure of bovine serum albumin (BSA), lysozyme (Lyz), and immunoglobulin G (IgG) were studied in 1:2:*n* choline chloride:glycerol:water (1:2:*n* ChCl:Glyc:H_2_O), with *n* = 0.4 to 20 mole-to-mole ratio, that is, from 2.2 to 53.4 wt % H_2_O. Similarly, the behavior of Lyz in 1:2:*n* choline chloride:urea:water (1:2:*n* ChCl:Urea:H_2_O), with *n* = 0.4 to 20 mole-to-mole ratio, that is, from 2.7 to 58.1 wt % H_2_O, was investigated to provide a comparison of the effect caused by the DES. We decided to use the 1:2:n ChCl:Glyc:H_2_O and 1:2:*n* ChCl:Urea:H_2_O solvents since their physicochemical properties, molecular structure, and DES–water interactions have been well resolved, covering the full range of hydration through experimental and computational methods.^12,45−47^ Similarly, BSA provides a characteristic overall conformation and has been extensively characterized in the native state and under different conditions of chemical and thermal stress,^35,51−55^ constituting a good proxy to understanding the effect of the DES hydration on protein folding, self-association equilibrium, and stability. Thus, BSA conformation and stability were investigated in 1:2:*n* ChCl:Glyc:H_2_O using small-angle neutron scattering (SANS), fluorescence spectroscopy, and temperature-dependent CD. The behavior of the proteins in the neat DESs and in an aqueous buffer (10 mM, pH 7 sodium phosphate buffer, herein referred to as the native state) provides the baseline comparisons to the results presented here. Notably, the residual water content was measured at *ca.* 0.37 wt % H_2_O (*n* = 0.065) in 1:2 ChCl:Glyc and 0.24 wt % H_2_O (*n* = 0.034) in 1:2 ChCl:Urea. These are expected values of the reported remanent water content in these DESs even after extensive drying.^12,45,46,56−58^ Thus, we refer to these as neat DESs. The equivalences of the water content in wt % and mol % are presented in Table S1. For simplicity and due to the similarity in the hydration levels between the two DESs systems, we will use the water contents of the 1:2:n ChCl:Glyc:H_2_O system to label the data throughout the article unless otherwise stated.

## Results

### Nonlinear Structural Transitions

**Figure 1:**
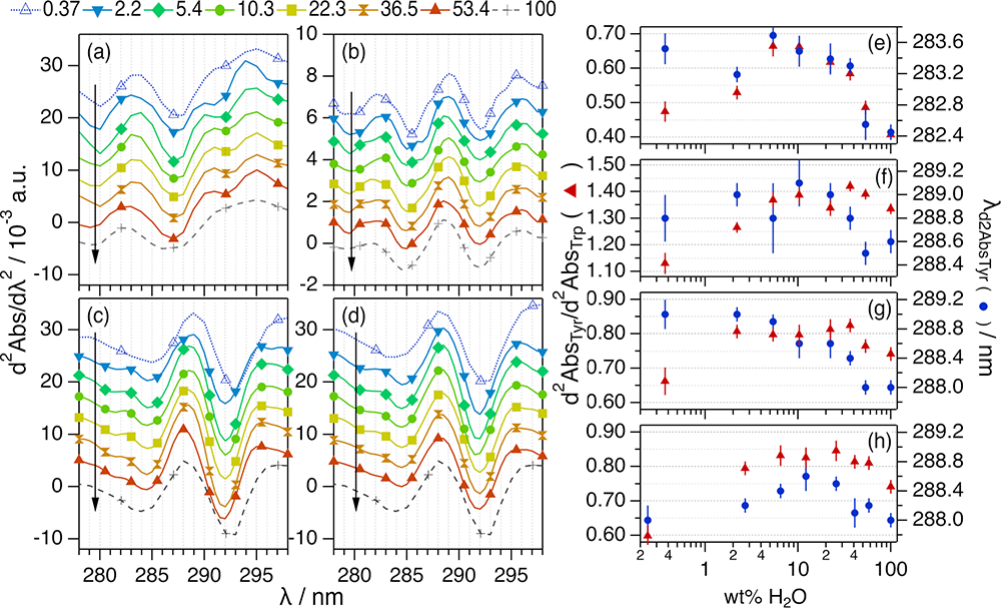
Second-derivative UV–vis spectra for (a) 100 μM BSA, (b) 20.4 μM IgG, and (c) 206 μM Lyz in 1:2:n ChCl:Glyc:H_2_O; (d) 224 μM Lyz in 1:2 ChCl:Urea:H_2_O at different DES hydrations expressed in wt % H_2_O in the solvent. The data and results for the proteins in aqueous buffer (100 wt % H_2_O) are presented for comparison. Data have been offset for clarity by (a,c,d) +0 (100 wt % H_2_O) +0.004, +0.008, +0.012, +0.016, +0.020, +0.024, and +0.028 (0.37 wt % H_2_O) and (b) +0 (100 wt % H_2_O), +0.001, +0.002, +0.003, +0.004, +0.005, +0.006, and +0.007 (0.37 wt % H_2_O). Hydration increases in the direction of the arrows. (e–h) present the results derived from the analysis of the spectroscopy data of the proteins, λ_d^2^Abs_Tyr__ and d^2^Abs_Tyr_/d^2^Abs_Trp_, in DES at different hydration levels. Where not seen, error bars are within the markers.

Initially, the solvation environment of the aromatic amino acids was investigated using UV–vis spectroscopy. To extract detailed information about the environment of the protein chromophores (e.g., solvent exposure), the second-derivative UV–vis spectra (d^2^Abs/dλ^2^) were calculated to resolve the changes in the signal of the peak centered at *ca.* 280 nm, from which the contributions of the tyrosine (Tyr, *ca.* 285 nm) and tryptophan (Trp, *ca.* 295 nm) sides chains can be discerned.^59^ Thus, the Tyr peak position (λ_d^2^AbsTyr_) and the ratio between the amplitudes of the Tyr and Trp peaks (d^2^Abs_Tyr_/d^2^Abs_Trp_) were determined. These data and results are presented in Figure 1. The full UV–vis absorption spectra for the different systems are presented in Figure S1. For further details on the analysis of these data, see the Supporting Information.

In neat and hydrated DESs, the proteins present the characteristic protein absorption spectrum with a peak centered at *ca.* 280 nm (Figure S1), similar in shape and intensity to the aqueous buffer, indicating the capacity of the neat and hydrated DESs to solubilize the proteins used in this study. The second-derivative UV–vis spectrum (d^2^Abs/dλ^2^) enables accessing the changes occurring in the molecular environment of the protein chromophores with the advantage of increased resolution compared to the relatively featureless UV–vis spectrum.^59^ From the results, it is observed that the features in the second-derivative UV–vis varied when changing the level of DES hydration; this qualitatively confirms changes in the amino acid environment and that the extent of those depends on the water content. The characteristic parameters of second-derivative UV–vis spectra are very sensitive to changes in the Tyr environment (peak at *ca*. 287 nm) and, to a minor extent, in the Trp residue (peak at *ca.* 295 nm), where an increase in d^2^Abs_Tyr_/d^2^Abs_Trp_ and a bathochromic shift in λ_d^2^Abs_Tyr__ relate to the exposure of the aromatic residues to a more polar environment.^59^

Generally, a dome-shaped transition is observed in the λ_d^2^Abs_Tyr__ and d^2^Abs_Tyr_/d^2^Abs_Trp_ for all systems investigated here (see Figure 1e–h). Three regimes can be differentiated: (i) At low hydration levels, the characteristic parameters gradually increase to their maxima around 4–8 wt % H_2_O; (ii) when the water content is increased between *ca.* 10 and *ca.* 40 wt % H_2_O, the value of d^2^Abs_Tyr_/d^2^Abs_Trp_ gradually decreases and a hypsochromic shift in λ_d2AbsTyr_ is observed; and (iii) above *ca.* 40 wt % H_2_O, the spectral parameters become similar to those in native conditions. Interestingly, the λ_d^2^Abs_Tyr__ and d^2^Abs_Tyr_/d^2^Abs_Trp_ in neat DESs reach similar magnitudes to those in aqueous buffer in some cases.

**Figure 2:**
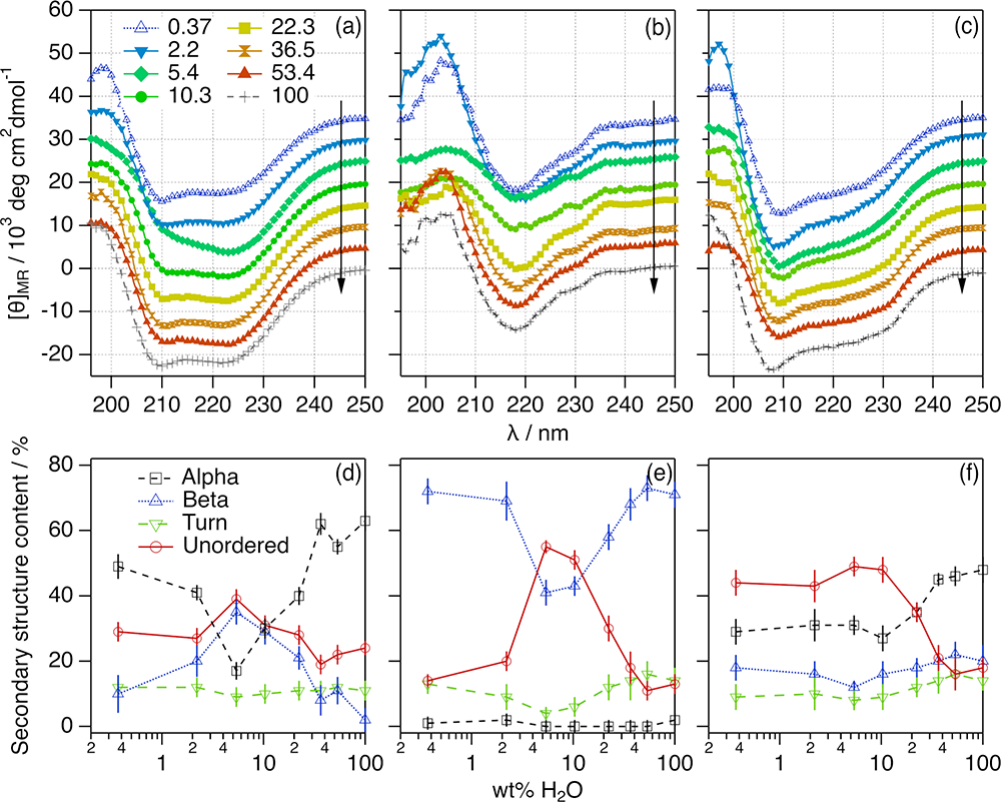
Mean residue ellipticity of (a) 59 μM BSA, (b) 20.4 μM IgG, and (c) 206 μM Lyz in ChCl:Glyc:H_2_O at different hydration levels. The data and results for the proteins in aqueous buffer (100 wt % H_2_O) are presented for comparison. Data have been offset for clarity by +0 (100 wt % H_2_O), +5 × 10^3^, +10 × 10^3^, +15 × 10^3^, +20 × 10^3^, +25 × 10^3^, +30 × 10^3^, and +35 × 10^3^ (0.37 wt % H_2_O). Hydration increases in the direction of the arrows. Hydration-dependent changes in the content of secondary structure motifs of (d) BSA, (e) IgG, and (f) Lyz were determined from the far-UV CD spectra. These values correspond to the average fitted values from three independent CD spectra. Where not seen, error bars are within the markers.

These transitions in the UV–vis spectral features are commonly associated with conformational changes in the protein, correlated to the exposure of buried aromatic residues that arises from a change in the amino acid milieu.^59^ To study the origin of those variations, the secondary structure of the proteins was investigated using far-UV CD measurements. The resulting CD spectra were analyzed to determine the population of each secondary structure motif in the protein using the software BeStSel in the wavelength range between 200 and 250 nm.^60^ The results are presented in Figure 2.

The proteins investigated provide a detailed comparison of the impact of DES hydration on specific folding motifs. In the native state, BSA is predominantly populated by α-helices with no β-sheet content,^61^ IgG folds into β-sheets with marginal amounts of α-helices,^62^ and Lyz contains populations of both ordered motifs (Tables S3–S5).^63^ Our results confirm that the hydration of the DESs induces changes in the secondary structure of the proteins, as observed in the spectral features of the CD data. Notably, the deconvolution of the CD spectra shows a general trend for the three proteins in 1:2:*n* ChCl:Glyc:H_2_O, where several transitions can be observed at different hydration regimes: (i) at low hydration (below *ca.* 8 wt % H_2_O), the proteins lose a certain amount of the main ordered secondary structure motif and the content of unordered structure increases; (ii) when water is increased within an intermediate hydration regime (between *ca.* 10 and *ca.* 40 wt % H_2_O), the ordered secondary structure of the proteins is gradually retrieved and the unordered structure decreases; and (iii) at high hydration (above *ca.* 40 wt % H_2_O), the native secondary structure is retrieved (within the error).

Interestingly, hydration affects differently to each protein and changes are particularly pronounced for BSA and IgG. In the case of BSA, a relative decrease of 0.78-fold in the α-helix content is observed in 1:2 ChCl:Glyc, accompanied by a subtle increase in the β-sheet and unordered content compared to those in the native state. At 5.4 wt % H_2_O, the protein loses 0.27-fold of the α-helix content, and the β-sheet and unordered contents increase by 17-fold and 2-fold, respectively. Finally, a similar secondary structure (within the error) to the BSA native state is observed above 36.5 wt % H_2_O. This suggests that DES hydration induces the unfolding of the secondary structure of BSA, with a characteristic α-helix-to-β-sheet transition, as previously observed upon albumin unfolding.^64^ For IgG, similar transitions are observed but affect the secondary structure motifs differently. In this case, the secondary structure population of the antibody is practically the same in 1:2 ChCl:Glyc as in aqueous buffer. However, the addition of water in the low hydration regime (5.4 wt % H_2_O) induces a 0.58-fold decrease in the population of β-sheets and a 4.2-fold increase in the content of unordered structures. This unfolding is again gradually reverted with increasing hydration. Changes for Lyz follow the same trends, although they are less pronounced. The low hydration unfolding is associated with a 0.6-fold decrease of the α-helix and β-sheet content and a 2.4-fold increase in the amount of unordered secondary structure. Also, the content of turn motifs in the proteins (*ca.* 10% of the total) hardly changes upon hydration.

It should be noted that the strong optical absorption of 1:2 ChCl:Urea in the far-UV region does not allow measuring the CD signal below 210 nm, even when using the narrow path cuvette (0.1 mm). Therefore, no quantitative estimation of the secondary structure of Lyz in 1:2:*n* ChCl:Urea:H_2_O can be performed with the available data at low hydration (Figure S2). However, some meager analysis was performed for the system at high hydration (Table S6), where the solvent is known to behave as an aqueous solution of the DES components.^45^ Our results confirm that the urea-based DES at high hydration (>40 wt % H_2_O) impacts protein structure to a larger extent than the glycerol analogues. This results in a decrease of the ordered secondary structure with decreasing water content in this hydration regime (e.g., 0.5-fold decrease in the α-helix content in 40.9 wt % H_2_O compared to the native secondary structure). The interaction of freely diffusing urea, a known potent denaturant, with Lyz is possibly the origin of this structural change; it has been shown that the disruption of the DES structure upon hydration enables specific interactions between the protein and the DES constituents.^65,66^ Still, it is observed that the native structure of the protein changes with hydration in 1:2:*n* ChCl:Urea:H_2_O and, considering the results from the UV–vis characterization of the system, these are likely to be non-monotonic.

Thus, a general mechanism seems to govern the amino acid environment and local folding of the proteins in hydrated DESs. Three particular regimes are observed with increasing hydration: (i) a low hydration regime, where water contributes to disrupting the structure of the protein, (ii) an intermediate hydration regime, where the protein gradually retrieves its native local fold, and (iii) a high hydration regime, where the native structure of the proteins is fully regained despite the high remanent amount of DES in the solvent.

### Protein Conformational Landscape

**Figure 3:**
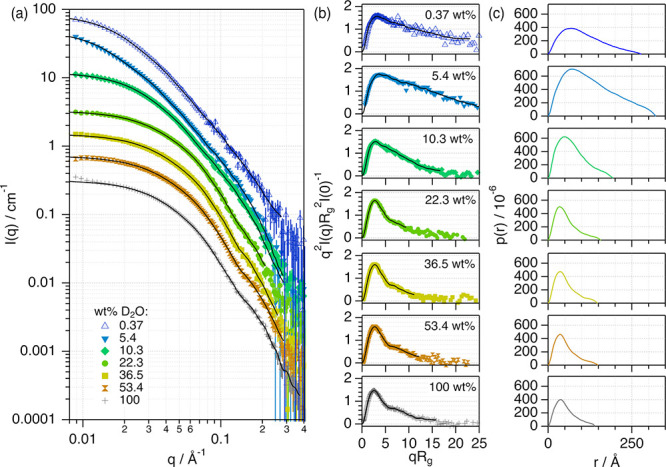
(a) SANS data and models of 55 μM BSA in 1:2:*n* d_9_-ChCl:d_8_-Glyc:D_2_O at different water contents in the solvent expressed in wt % D_2_O. (b) Normalized Kratky representation of the SANS data and models and (c) pair-distance distribution functions of the protein in different solvents. The data and results for BSA aqueous buffer (100 wt % D_2_O) are presented for comparison. The models obtained from the indirect Fourier transform analysis are presented as solid black lines. The legend for the data is presented in panels (a,b). Data and models in (a) have been offset for clarity by ×1 (100 wt % D_2_O), ×2, ×4, ×8, ×16, ×32, and ×64 (0.37 wt % D_2_O). Where not seen, error bars are within the markers.

To relate the observed re-entrant changes above to the protein’s hierarchical structure, SANS experiments were performed on 55 μM BSA (3.66 mg/mL, average concentration) to study the effect of 1:2:*n* ChCl:Glyc:H_2_O at different levels of hydration. Deuterium-labeled solvents were required to gain contrast in SANS experiments (1:2:n d_9_-ChCl:d_8_-Glyc:D_2_O). Previous investigations have shown that solvent deuteration has a minimal impact on the behavior of this protein in DESs.^35^ Also, SANS does not cause any radiation damage to the sample. Thus, we preferred this method to other alternatives, such as synchrotron-radiation small-angle X-ray scattering, which is known to cause beam damage due to the high electron density of the DESs.^67^ The data, models, and pair-distance distribution functions (*p(r)*) of the scatterer are presented in Figure 3.

Our SANS data show that the signal from BSA significantly changes when the water content varies in the DES. These differences are highlighted in the normalized Kratky plots, which represent the normalized scattering intensity, *q*^2^*I*(*q*)*R*_g_^2^*I*(0)^−1^, against *qR*_g_, where *I*(*q*) is the scattered intensity, *I*(0) is the extrapolated scattering intensity at angle zero, *q* is the momentum transfer vector, and *R*_g_ is the radius of gyration of the scatterer. In this plot, the decay at high qR_g_ relates to the folding state of the protein (see Figure 3b).^68^ The bell shape of the Kratky plot for BSA in 1:2 ChCl:Glyc shows that the decay at high qR_g_ (>5) is less pronounced in neat DES than in the aqueous buffer. This confirms that the protein changes conformation but retains a certain degree of globularity in the DES compared to aqueous buffer.^35^ In the hydrated DESs, different regimes are observed: (i) at a low water content (5.4 wt %), the protein swells as reflected by the change in the negative slope at high qR_g_; (ii) when the water content is increased to 10.3 wt % D_2_O, the slope of the Kratky representation suggests that the protein folds into a more compact conformation compared to that at low DES hydration; and (iii) above 36.5 wt % D_2_O, the shape of the Kratky plot is similar to that of the protein in the native state.

**Figure 4:**
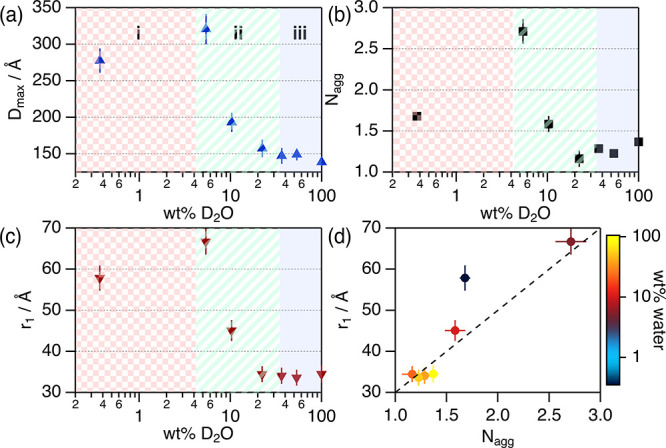
Parameters derived from the analysis of the SANS data of 55 μM BSA in 1:2:n d_9_-ChCl:d_8_-Glyc:D_2_O at different water contents: (a) *D*_max_, (b) *N*_agg_, and (c) *r*_1_ as a function of wt % D_2_O. (d) Parametric plot of the variation of *r*_1_ vs *N*_agg_ at different levels of solvent hydration. The water content in (d) is represented following the color scale. The dashed line is a guide for the eye of the trend described by the results in hydrated DES.

The indirect Fourier transform method was used to calculate the *p(r)*, representing a histogram of the distances between two points within the scatterer, to extract detailed structural information.^69−71^ From the p(r), the characteristic structural parameters of BSA were calculated: the maximum dimension of the protein, *D*_max_, the folded state of the monomer parameterized as the position of the first peak in the p(r), *r*_1_, and the protein self-association parameterized as the aggregation number, *N*_agg_ (Figure 4). Details on the data analysis and the determination of these parameters are presented in the Supporting Information.

In the native state, the conformation of BSA in solution is a mixture of folded (globular) monomers and dimers in a dynamic equilibrium.^52,72^ Our characterization of BSA in aqueous buffer agrees with the previously reported conformation of this protein in the native state, confirming that the selected buffer does not affect the structure of the protein. BSA’s calculated *p*(*r*) in the different solvents confirms that the neat DES promotes conformational changes (see Figure 4a). We observe that the *D*_max_ of BSA in 1:2 ChCl:Glyc (280 ± 17 Å) doubles that of the protein in aqueous buffer (138 ± 5 Å).^73^ The size change confirms a shift in the native BSA self-association (monomer–dimer equilibrium) toward a higher population of oligomers in the neat DES, potentially a monomer–dimer–tetramer equilibrium.^72^ This behavior has been previously reported for aqueous solutions of BSA at high ionic strength, where the protein self-associates into tetramers (maximum diameter *ca.* 300 Å) due to an increase in protein–protein attraction.^53,54^ The *r*_1_ value increases when BSA is solvated in 1:2 ChCl:Glyc (57.8 ± 3.1 Å) compared to that in the aqueous buffer (34.5 ± 2.1 Å). This larger value, together with the shape of the Kratky plot, suggests that the BSA monomer is partially folded in the neat DES compared to the fully folded native folded state.

The addition of water to 1:2 ChCl:Glyc induces changes in the conformational state of BSA in both the folding state and self-association, even at low water content. Importantly, these changes are non-monotonic: (i) An increase in *D*_max_, *r*_1_, and *N*_agg_ is observed at 5.4 wt % D_2_O compared to the neat DES, showing that BSA is further unfolded and increases self-association. (ii) At 10.3 wt % D_2_O, the protein becomes more compact and the self-association decreases but remains in a partially folded, self-associated state. (iii) At 36.5 wt % D_2_O and above, the protein retrieves its native folding and self-association equilibrium. From the results presented in the parametric plot (Figure 4d), we observe that the protein populates different conformational states depending on the water content: there is a linear correlation between the monomer structure (*r*_1_) and self-association (*N*_agg_) in hydrated DES; however, the protein does not follow these conformational features in neat DES. Therefore, water must play a significant role in the behavior of the protein, possibly connected to the changes in the nanoscale organization of the solvent.^45−47^

**Figure 5:**
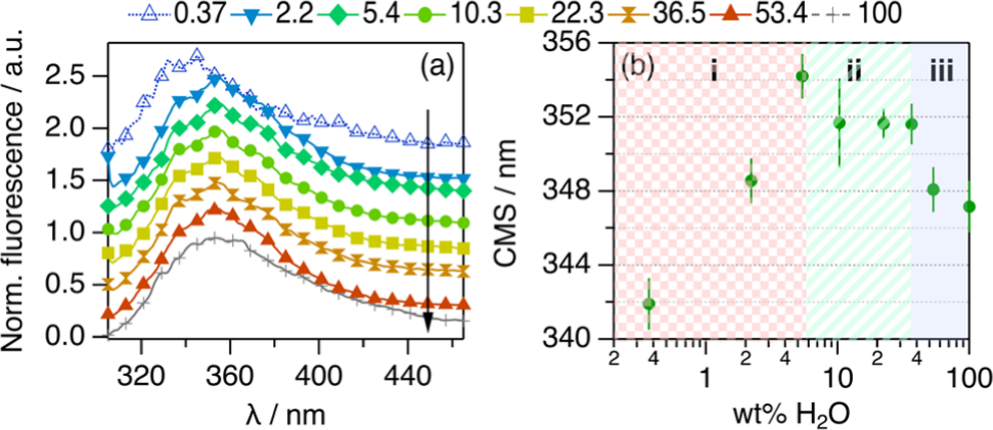
(a) Trp emission spectra from 10 μM BSA in 1:2:n ChCl:Glyc:H_2_O at different hydration levels expressed in wt % H_2_O in the solvent. The data and results for BSA aqueous buffer (100 wt % H_2_O) are presented for comparison. Data in (a) have been offset for clarity by +0 (100 wt % H_2_O), +0.25, +0.50, +0.75, +1.00, +1.25, +1.50, and +1.75 (0.37 wt % H_2_O). Hydration increases in the direction of the arrows. (b) CSM results derived from the analysis of the Trp emission spectra between 305 and 380 nm.

BSA has two Trp residues buried at the hydrophobic pockets of domains I and II.^74^ As such, intrinsic Trp fluorescence spectroscopy can be used to determine the degree of solvent accessibility to the hydrophobic core associated with the conformational changes. From the experimental data, the center of spectral mass (*CSM*) of the Trp emission spectra was determined at each hydration level. The CSM accounts for the position and intensity distribution across the emission peak; thus, it can be used to identify changes in the environment of the Trp residue. These results are shown in Figure 5.

The Trp emission spectra and CSM show the same characteristic transitions observed in the UV–vis results (see Figure 5b): (i) a bathochromic shift is observed at low water content, (ii) this is followed by a hypsochromic shift when hydration is increased within intermediate water contents, and (iii) the CSM plateaus at high hydration levels at a similar value to that in the native state. These changes could be attributed to variations in the polarity of Trp’s solvation milieu or specific Trp–DES interactions. For instance, it has been shown that the choline cation interacts with the exposed Trp residues of Lyz in choline-based DESs.^50,65^ However, previous investigations showed that the emission wavelength of L-Trp in 1:2 ChCl:Glyc increases, at least, by 2 nm compared to that in aqueous buffer.^75^ Here, it is observed that the change in the emission of the Trp residues of BSA in 1:2 ChCl:Glyc is opposite to that of the isolated amino acid. As most of these residues are buried in the hydrophobic core of the protein in the native state,^51,55,74^ these changes cannot be solely attributed to solvent exposure and must relate to conformational transitions occurring in the protein, where the Trp side chains are relocated inside the protein envelope. In contrast, the bathochromic shifts observed at low hydration potentially relate to an increase in the solvent exposure of Trp and a more pronounced unfolding. Similarly, the hypsochromic shifts observed with further increasing hydration are associated with the refolding of BSA. Thus, these changes agree with the conformational transitions determined from the SANS data.

Notably, the boundaries of each defined region (i, ii, and iii) overlap between the different techniques (UV–vis, CD, SANS, and fluorescence). This confirms that protein concentration in the dilute regime and isotope substitution in the solvent have negligible effects on the behavior of the protein.

### Protein Thermal Stability

**Figure 6:**
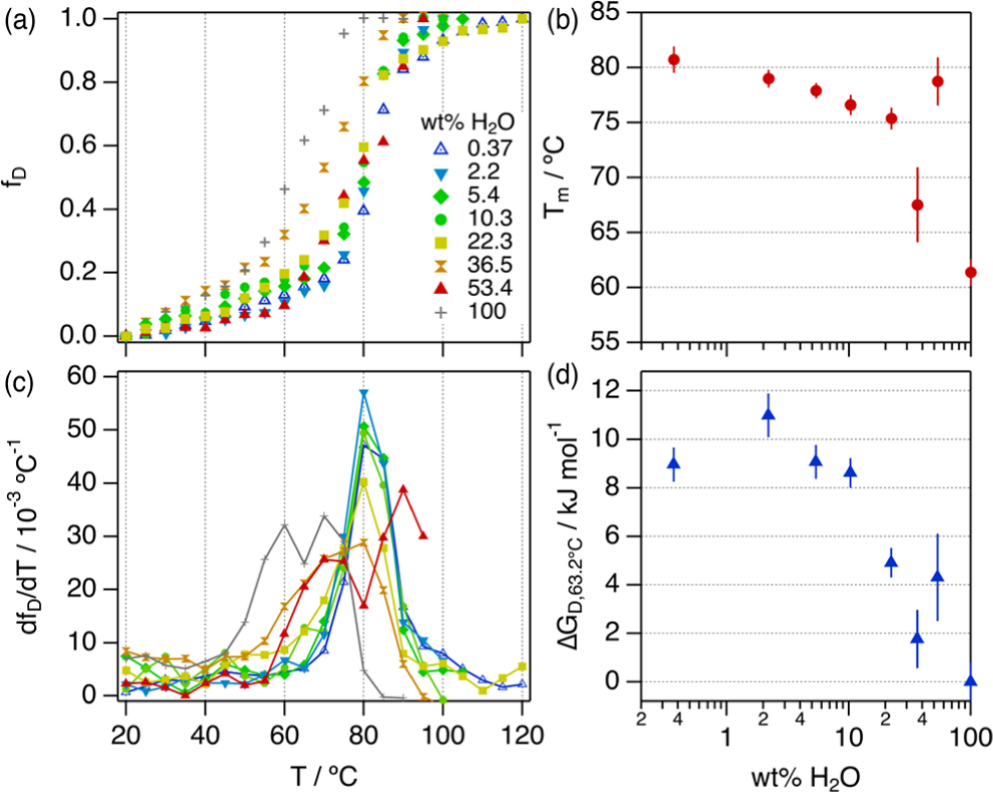
(a) Equilibrium fraction denatured and (c) first derivative of the fraction denatured for BSA in 1:2:n ChCl:Glyc:H_2_O at different hydration levels as a function of the temperature. The legend for the data is presented in (a). (b) Half denaturation temperature and (d) free energy of denaturation for BSA in neat and hydrated DESs at 63.2 °C. Error bars represent the standard deviation to the observed mean from three repeats performed on independent samples. Where not seen, error bars are within the markers.

Considering the conformational transitions of BSA, temperature-dependent CD measurements were performed to determine whether solvent hydration affects the thermal stability of the protein (see Figure S4). Increasing the temperature from 20 to 120 °C, the spectra showed a progressive reduction of the negative intensity at 208 and 222 nm. This change is associated with the loss of the defined secondary structure, α-helix and β-sheet, and the concomitant increase in the unordered content. To rigorously quantify the denaturation transition, the equilibrium fraction denatured (*f*_D_) of BSA at each temperature was calculated from the variation of the spectral signal at 222 nm (see the Supporting Information for details on these calculations). The f_D_ as a function of temperature for each system is presented in Figure 6.

The f_D_ gradually increases when the sample temperature increases with a sharp transition at the thermal denaturation of the protein. Notably, the temperature at which the transition occurs is different for each hydration level, as observed in the first derivative of the fraction denatured (see Figure 6b). The latest transition occurs for BSA in neat ChCl:Glyc, and the earliest transition is observed for BSA in aqueous buffer. The solvents with hydrations at and below 22.3 wt % H_2_O show a sharp sigmoidal transition in f_D_ and a single peak in the d*f*_D_/d*T*. This trend confirms that the thermal denaturation process at low hydration essentially follows a two-state unfolding mechanism.^2,76,77^ On the contrary, it is observed that BSA in aqueous buffer (100% H_2_O) and hydrated DES with 53.4 wt % H_2_O shows two peaks in the first derivative, potentially associated with a transition involving unfolding intermediates. More complex is the broad transition observed for BSA in the solvent containing 36.5 wt % H_2_O, which can be attributed to a multiple-state denaturation mechanism.^78^

Using a two-state transition model, the characteristic thermodynamic parameters associated with the unfolding process, that is, the half-denaturation temperature (*T*_m_) and the free energy of denaturation at 63.2 °C (Δ*G*_D_), were determined (see Figures 6b,d and S5).^76,79^ For the system at high water content (36.5, 53.4, and 100 wt % H_2_O), the system was approximated as a two-state transition, provided that the shape of the *f*_D_ does not show any sharp features in the transition region that can be attributed to the accumulation of unfolding intermediates around the *T*_m_ (e.g., molten globules and aggregates).^76^*T*_m_ is the highest in neat 1:2 ChCl:Glyc with a 31.5% ± 0.4% increase relative to *T*_m_ in aqueous buffer. When the DES is hydrated, *T*_m_ gradually decreases with increasing hydration between 2.2 and 36.5 wt % H_2_O. Surprisingly, this trend is reversed at 53.4 wt % H_2_O as the *T*_m_ increases by 28.3% ± 1.9% compared to the aqueous buffer. The stabilizing effect promoted by the presence of the DESs is also reflected in Δ*G*_D_. BSA in hydrated DESs shows a higher Δ*G*_D_ value than that in aqueous buffer (Δ*G*_D,63.2°C_ = 0 kJ/mol) in all cases, indicative of increased stability. In particular, the solvents with the lowest level of hydration, that is, between 0.37 and 10.3 wt % H_2_O, show the largest gain in stability with a Δ*G*_D_ of ca. 9 kJ/mol. At higher hydration, the variation becomes again non-monotonic as the hydrated DES with 53.4 wt % H_2_O shows a higher Δ*G*_D_ than that with 36.5 wt % H_2_O.

These results prove that the neat and hydrated DESs induce a stabilizing effect against the thermal denaturation of BSA. Two different stabilization pathways are observed. (1) At low DES hydration (<22.3 wt % H_2_O), BSA unfolding follows a two-state mechanism, where the protein evolves from the partially folded conformation to a denature state upon heating. Importantly, BSA shows the highest stability in the conditions investigated here in this hydration regime. (2) At high DES hydration (>36.5 wt % H_2_O), BSA follows a non-two-state transition. This is similar to BSA in aqueous buffer, where protein aggregation upon heating restricts the refolding of early unfolded protein and generates denaturation intermediates.^78^ Interestingly, the thermal stabilization at 53.4 wt % H_2_O potentially arises from an indirect protection mechanism as the conformation and solvation of the protein are very similar to that in the native state. However, this demonstrates that even dilute 1:2 ChCl:Glyc is remarkably capable of protecting the protein against thermal unfolding.

### Conformational and Functional Recovery

**Figure 7:**
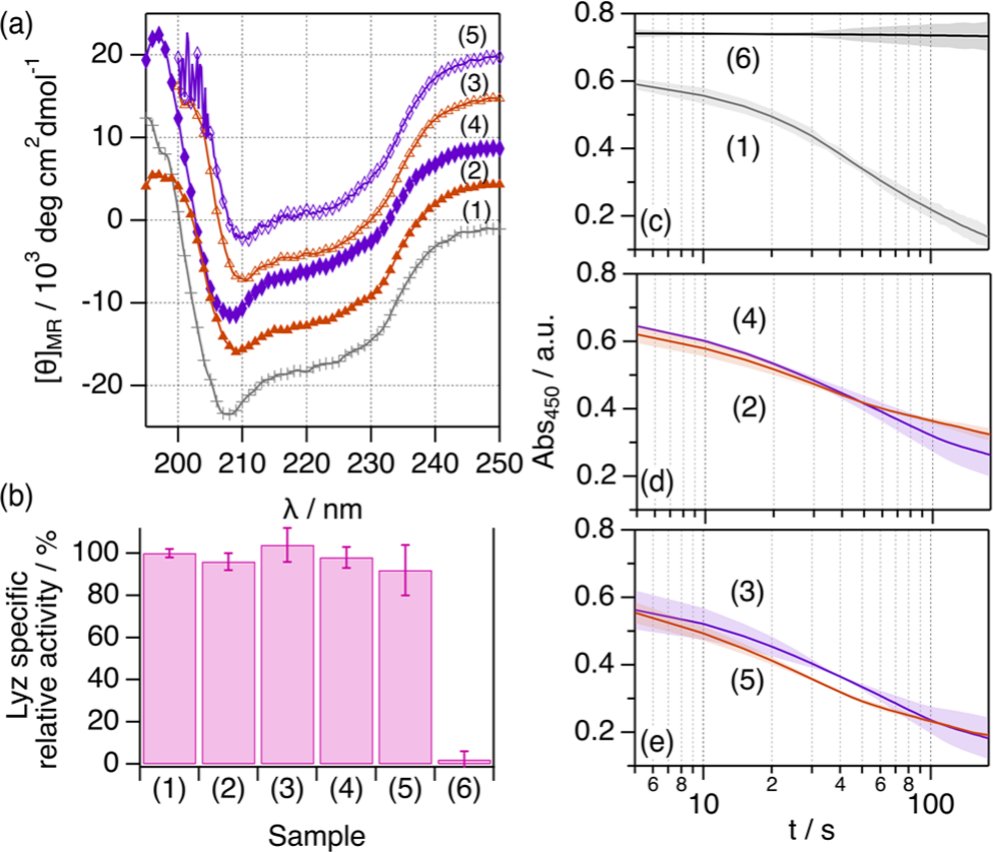
(a) Mean residue ellipticity for 107 μM Lyz in (1) aqueous buffer, (2) 1:2:20 ChCl:Glyc:H_2_O with 53.4 wt % H_2_O, (3) rehydrated 1:2:20 ChCl:Glyc:H_2_O to 53.4 wt % H_2_O, (4) 1:2:20 ChCl:Urea:H_2_O with 58.1 wt % H_2_O, and (5) rehydrated 1:2:20 ChCl:Urea:H_2_O to 58.1 wt % H_2_O. Data have been offset for clarity by +0 (aqueous buffer), +5 × 10^3^, +10 × 10^3^, +15 × 10^3^, and +20 × 10^3^ (rehydrated 1:2:20 ChCl:Urea:H_2_O). (b) Specific relative activities of Lyz in the different samples. Sample (6) corresponds to the data recorded for a *Micrococcus lysodeikticus* suspension in the absence of Lyz. The sample activities (IU mg^–1^ of Lyz) were normalized to the activity of Lyz in aqueous buffer. (c–e) Change in the absorbance at 450 nm of a *Micrococcus lysodeikticus* cell suspension upon the addition of Lyz for the spectrophotometric determination of Lyz activity in the different samples. Error bars represent the standard deviation to the observed mean from three repeats.

To test whether the unfolding observed at low hydration, that is, regime i, led to the formation of trapped unfolded states or unfunctional aggregates, we study the reversibility of the system upon rehydration to regime iii. After stabilizing Lyz in the hydrated 1:2:1 ChCl:Glyc:H_2_O (5.4 wt % H_2_O) for 24 h and 1:2:1 ChCl:Urea:H_2_O (6.5 wt % H_2_O), where the protein remains unfolded (see Figure 1), water was added to the sample to reach 53.4 wt % H_2_O and 58.1 wt % H_2_O for the glycerol-based and urea-based DESs, respectively. At this degree of hydration, the protein is expected to regain its folded structure and activity if the unfolding at low hydration is reversible. These samples are labeled as rehydrated from hereon. Far-UV CD were used to study the secondary structure of Lyz, and activity assays were performed to study Lyz function under the different conditions.^33^ The results from the characterization are shown in Figure 7.

The estimation of the secondary structure content in the rehydrated sample shows a recovery of the local folding of Lyz, with similar populations within the error to those of the sample at high hydration (Table S8). Again, the sample containing the rehydrated urea-based DES shows a slight drop in the population of ordered structures, as seen in the hydrated 1:2:20 ChCl:Urea:H_2_O. Also, the addition of Lyz to the cell suspension prompts a rapid decrease in the absorbed intensity in all cases, associated with the bactericidal action of the protein. Note that the absorbed intensity of the cell suspension does not decrease in the absence of Lyz. From the initial rate of the lysis, the activity of the enzyme in the rehydrated DESs was calculated and compared to that of the systems at high hydration and to that of the enzyme in aqueous buffer. Our results show that the normalized activity values in the rehydrated samples are similar to those of Lyz in DESs at high hydration and in native conditions. Thus, the enzyme is fully active in these samples. This confirms that the unfolding observed at low water content can be reversed through the addition of water, that is, rehydration, accompanied by the recovery of the protein function.

## Discussion

The re-entrant character of protein behavior suggests a complex interplay between the DES components and the protein. As the addition of water should increase the solvophobic effect and, thus, the folding tendency of the protein, an alternative mechanism is required to explain the results gathered here, where hydration cannot solely explain the behavior of the proteins.^9^ From these results, we propose a mechanism for protein behavior in neat and hydrated DESs.

In neat DESs, the protein undergoes small changes in the secondary structure, which alter the overall conformation of the protein to a partially folded state compared to the native folding. Molecular dynamics (MD) simulations showed that the changes are attributed to forming of a solvation matrix stabilized by hydrogen bond interactions between the protein residues and the DES constituents.^51^ These solvation and stabilization mechanisms were previously observed for several proteins, such as α-chymotrypsin, lysozyme, and subtilisin in glycerol-based DESs.^35,36,42,80^ A similar protective effect has been reported in glycerol, which also enhances protein stability.^81^ However, our results show that glycerol alone does not cause significant conformational changes in BSA (Figure S3, Table S7). Thus, the changes observed for the proteins in DESs must come from contributions of the ionic species in the solvent, that is, choline chloride. Furthermore, the solubilization of antibodies in choline-based ionic liquids was shown to induce conformational transitions, which could enhance the stability under certain conditions.^82,83^ This solubilization mechanism hinders fluctuations in the protein structure,^51,66^ and could also contribute to the enhanced thermal stability. These arrested dynamics could reduce the denaturation tendency despite the partially folded conformation of the protein.

Upon DES hydration, we observe a re-entrant behavior of the folding and solvation environment of the protein. We hypothesize that these changes are connected to the non-monotonic variations in the solvent nanostructure upon solvation,^45−47^ possibly involving non-specific (preferential solvation) and protein–DES-specific interactions, as previously observed using MD simulations of proteins in neat and diluted DESs.^51,65^ At low hydration levels (i, < *ca.* 10 wt % water) in 1:2 ChCl:Glyc, water becomes part of the DES network by displacing Cl^–^ anions. The resulting solvent arrangement could be more prone to interact with the proteins through hydrogen bonds and electrostatic interactions, affecting their electrostatic landscape and promoting further loss of folding compared to the more charge-balanced neat DESs. Concomitantly, the pronounced exposure of hydrophobic residues increases patchy attractive interactions and promotes the formation of more transient oligomers at low hydration levels.^52,84^ When the water content is increased between 10 and 40 wt % (ii), water molecules dissociate the DES components into smaller clusters and fill interstitial spaces. This potentially weakens the interaction between the protein and the DES components, and water starts to solvate the protein preferentially, as observed for other small solutes such as thiocyanate.^85^ The weakened DES–protein paired interaction results in the gradual refolding of the protein in this solvation regime. At high hydration levels (iii, > *ca.* 40 wt % water), the DES components are fully dissociated and freely diffusing. As a result, we observe that the effects of the DES on the behavior of the protein vanish, and water preferentially solvates the proteins.^85^ This could parallel the observations of specific solvation in glycerol/water mixtures, where water preferentially solvates the protein above 40 wt % water.^86^ It is also important to keep in mind that the dissociated species in regime iii could effect protein behavior,^45^ as shown for the urea-based hydrated DES.

Unlike the effect of chemical denaturants, which reduce the stability of proteins in connection to the loss of a folded conformation,^55^ the glycerol-based DES and DES–water mixtures enhance BSA’s stability in a partially folded state. This behavior has been reported for molten globules in aqueous buffers, ionic liquids, and chemically engineered proteins solubilized in different media.^10,77,79,87−89^ However, the enhanced stability is not a general case in neoteric solvents as hydrated ionic liquids reduce the stability of the green fluoresce protein.^10^ These effects could be intrinsically related to the properties of the cybotactic region, as the high viscosity of a choline-based DES was shown to control the folding of DNA and the stabilization of kinetically trapped non-native folds.^90^ This is a similar mechanism to that observed in organic solvents, where the solubilization of proteins in anhydrous organic media significantly enhances the stability of proteins due to the restrictions in protein conformational mobility.^6,7,48^ However, adding water to the system facilitates conformational changes in the protein, as it happens in the DESs, which gradually decreases this stability enhancement. The addition of water to the hydrated systems can also be used to shift the conformational state of the protein. The unfolded proteins at low hydration can be refolded via rehydration, also leading to recovery of the protein function at the water content. Notably, the protein does not show any signs of aggregation at any hydration level, possibly attributed to the presence of electrostatic colloidal repulsion in the neat and hydrated DESs despite the high ionic strength of the solvent.^91^

Therefore, the behavior of these proteins in neat and hydrated DESs parallels that of other proteins in organic solvents and ionic liquids, where specific solvent–protein interactions and the formation of segregated solvent domains strongly correlate to the differences observed in protein conformation, stability, and function.^9,10,48,49^ DESs appear as promising candidates for enzymatic catalysis,^92^ biomolecule preservation,^33^ protein crystallization,^50^ and drug delivery,^83^ among others; these results will help in the rational development of pre-design solvents for optimized performance. In particular, the stabilization of antibodies, which is of great importance in pharmaceutical technology, makes DESs a potential alternative for the protection of these labile biomolecules in solution.^83^ In addition, future investigations involving MD simulations will help to reveal the molecular origin of the protein–solvent interactions, helping to boost those that enhance protein performance and stability.^38^ Notably, further developments are required to reliably incorporate biomolecules into DES-containing simulation boxes, where slight inaccuracies in the highly complex energy landscape of the solvent could override the fine energy balance that defines protein folding in these systems.

## Conclusions

In summary, we demonstrate that two model proteins and an antibody undergo several transitions with the hydration of 1:2 ChCl:Glyc and 1:2 ChCl:Urea following an overarching mechanism. These changes are non-monotonic and follow a re-entrant fashion: a folded or partially folded conformation is observed in neat DES and adding small amounts of water to the system promotes further unfolding. This trend is reversed with higher hydration as the native fold is gradually retrieved above the threshold of *ca.* 10 wt % water in the DESs. However, it takes *ca.* 40 wt % water to completely retrieve the native protein folding and solvation environment in glycerol-based hydrated DES. In contrast, the urea-based DES at high hydration affects protein structure but still to a lower extent than this solvent at low hydration. This is potentially attributed to the presence of freely diffusing urea acting as a protein denaturant.

We also show that the changes induced by hydration affect the higher structure of proteins, as shown for BSA. Consequently, changes in the secondary structure correlate to transitions in the folded state and self-association of the protein. We also report that the solubilization of the protein in neat and hydrated 1:2 ChCl:Glyc prompts an increase in the thermal stability of BSA. The protein shows the highest stability at low hydration (<10 wt % water), but even the DESs in dilute conditions show a remarkable stabilizing effect against thermal denaturation. One of the interesting aspects of the conformational landscape in hydrated DESs is that transitions can be achieved with variations in the water content, for example, rehydration, leading to a fine control over the protein behavior and the stabilization of folding intermediates.

These results confirm that the conformation of the protein is strongly correlated to the hydration of DESs, where the changes in protein conformation, local folding, and solvation environment co-occur. In particular, we hypothesize that such transitions are interconnected in a way that the solvation environment of the protein, defined by non-specific preferential solvation and specific interactions between the protein and the solvent (either the DES components or water), controls the overall folding of the proteins. As the nanoscopic changes in DESs upon hydration seem to be ruled by a general mechanism, where water has a nonlinear de-structuring effect on the DES,^45−47^ the transitions reported here can potentially be extrapolated to other protein systems. Therefore, quantifying the water content of the system becomes imperative as unexpected changes in protein behavior may occur at different hydration levels or upon water adsorption due to the hygroscopic nature of DES.

## Experimental Section

1:2 choline chloride:glycerol, its deuterated analogue and 1:2 choline chloride:urea were prepared by mixing the components and heating at 60 °C under an argon atmosphere until a colorless, transparent liquid was formed. The proteins were incorporated into the neat DESs using a freeze-drying approach on an Epsilon 2-6D LSCplus from Martin Christ. The residual water content in the neat DESs was determined using Karl–Fischer titration and found to be 0.37 ± 0.10 and 0.24 ± 0.08 wt % water. Samples containing protein in hydrated DESs were prepared by mixing an aqueous protein stock solution with the DES and water or D_2_O to the required protein concentration and water content. Protein concentration was determined using an ND-1000 Spectrophotometer (Saveen Werner).

SANS experiments were performed on D22 at Institut Laue-Langevin under experiment number 8-03-1049.^93^ Reduced data were analyzed using the indirect Fourier transform method implemented in the GNOM software.^71^ CD measurements were performed on a Jasco J-715, and spectra were analyzed using BeStSel.^60^ Temperature-dependent CD data were collected using a JASCO J-1100 equipped with a Peltier sample stage. UV–vis spectroscopy measurements were performed on a Varian Cary 50 UV–Vis Spectrometer and a JASCO V-770 UV–vis/NIR Spectrophotometer. Fluorescence spectroscopy measurements were performed on a Cary Eclipse fluorescence spectrometer. Data are openly available at DOI:10.5281/zenodo.6341232 and 10.5291/ILL-DATA.8-03-1049.^93^

## Supporting Information Available

Materials and methods, UV–vis data of the proteins in the different solvents, populations of secondary structure motifs in hydrated 1:2:n ChCl:Glyc:H_2_O, circular dichroism of Lyz in 1:2:n ChCl:Urea:H_2_O, BSA conformation in glycerol with 10 wt % water, temperature-dependent CD spectra, and thermodynamic analysis of the temperature-dependent scans (PDF)
