# Supplementary material for: Hydration in Deep Eutectic Solvents Induces Non-monotonic Changes in the Conformation and Stability of Proteins
Source: J Am Chem Soc. 2022 Dec 16;144(51):23657–67. doi: 10.1021/jacs.2c11190 (PMC9801427; doi:10.1021/jacs.2c11190)
Supplement: Supplementary file 1 — ja2c11190_si_001.pdf [file ja2c11190_si_001.pdf]

Supporting information for:
Hydration in deep eutectic solvents induces non-monotonic changes in the conformation and stability of proteins

Adrian Sanchez-Fernandez,[a,b]* Medina Basic,[b] Jenny Xiang,[b] Sylvain Prevost,[c] Andrew J. Jackson,[d,e] Cedric Dicko,[f,g]
[a]Centro Singular de Investigación en Química Biolóxica e Materiais Moleculares (CIQUS), Departamento de Química Orgánica, Universidade de Santiago de Compostela, 15705 Santiago de Compostela, Spain.
[b]Food technology, Engineering and Nutrition, Lund University, Box 117, SE-221 00 Lund, Sweden.
[c]Institut Laue-Langevin, DS / LSS, 71 avenue des Martyrs, 38000, Grenoble, France.
[d]European Spallation Source, Lund University, Box 176, SE-221 00 Lund, Sweden.
[e]Divison of Physical Chemistry, Department of Chemistry, Lund University, Box 124, SE-221 00 Lund, Sweden.
[f]Pure and Applied Biochemistry, Department of Chemistry, Lund University, Box 124, SE-221 00 Lund, Sweden.
[g]Lund Institute of Advanced Neutron and X-ray Science, SE-223 70 Lund, Sweden.
*adriansanchez.fernandez@usc.es

Materials and methods

Materials

Bovine serum albumin (BSA, fatty acid free, >98%), lysozyme from hen egg white (Lyz, >98%), immunoglobulin G (IgG, >95%, four subclasses in natural abundance), choline chloride (ChCl, >99%), glycerol (Glyc, >99.5%%), urea (99.0-100.5%), sodium phosphate monobasic dihydrate (>99.5%), sodium hydroxide (>99%), sodium azide (NaN_3, >99.5%) and D_2O (99.9 %D) were purchased from Sigma and used as received. Choline-d_9 chloride (d-ChCl, 98 %D) and glycerol-d_8 (d-Glyc, 99 %D) were supplied by Cambridge Isotope Labs and used without further purification. Water used for sample preparation was of Milli-Q grade (resistivity 18.2 MΩ cm^{-1} at 25 ˚C).

The chemicals needed for the preparation of the deep eutectic solvents (DESs) were vacuum dried at 70 °C (ChCl and Urea) or 50 °C (Glyc) for 24 hours before preparation of the DESs. 1:2 ChCl:Glyc, 1:2 ChCl:Urea and the deuterated analogue 1:2 d_9-ChCl:d_8-Glyc were prepared by weighing the required amounts of each chemical into a round bottom flask, followed by continuous stirring and heating at 80 °C until a clear liquid was formed. After cooling, the DESs were equilibrated at room temperature in a desiccator for a minimum of two days before use. Once ready, the DESs were stored sealed and under a dry atmosphere to avoid water adsorption. The residual water in the neat DESs was measured using Karl-Fischer titration to an average water content 0.37 wt% and 0.24 wt% in 1:2 ChCl:Glyc and 1:2 ChCl:Urea, respectively.

The hydrated DES were prepared by mixing the required amount of 1:2 ChCl:Glyc or and 1:2 ChCl:Urea with Milli-Q water to obtain the following solvents with water contents: 1:2:n ChCl:Glyc:water with n= 0.4, 1, 2, 5, and 20, and 1:2:n ChCl:Urea:water with n= 0.4, 1, 2, 5, and 20. The corresponding molar and weight percentages are presented in Table S1. The same approach was used to prepare the deuterated solvent (1:2 d_9-ChCl:d_8-Glyc:D_2O) using D_2O.

Table S1. The water contents of the series of hydrated DES used in these experiments. Mol% water is defined as the $mol_{water}/mol_{total} \times 100$.

1:2:n ChCl:Glyc:H_2O		
n	wt% water	mol% water
0.065	0.37	2.12
0.4	2.2	11.8
1	5.4	25.0
2	10.3	40.0
5	22.3	62.5
10	36.5	76.9
20	53.4	92.1
1:2:n ChCl:Glyc:H_2O		
0.034	0.24	1.45
0.4	2.7	14.8
1	6.5	30.3
2	12.2	46.5
5	25.7	68.4
10	40.9	81.3
20	58.1	89.7

Phosphate buffer (10 mM, pH 7 in water) was used as reference for the protein state in aqueous buffer in the experiments presented here.

Sample preparation

Initially, protein stock solutions were prepared by adding the required amounts of BSA to water (Milli-Q or D_2O) for a protein concentration of *ca.* 1000 µM. Two- and ten-fold dilutions of the 1000 µM samples were conducted to obtain the stock solutions at lower concentrations, 500 and 100 µM respectively. Each protein solution was then centrifuged at 13 000 rpm for 1 min, followed by pipetting of the supernatant into new tubes to remove any aggregates from the solution.

Protein samples in neat DESs were prepared by mixing the required amount of DES and protein stock solution (non-buffered) in a glass vial. The concentration of protein was optimized for each experimental method to obtain the best signal-to-noise ratio. The added amount of protein stock solution was at least 20 times lower than the added amount of DES (v/v) to reach the required final protein concentration with the lowest possible amount of water prior drying. Subsequently, samples were freeze-dried using an optimized protocol as explained below. Control samples of the DES without protein were tested for water content upon drying using Karl-Fischer titration showing that minor variations in the water content (±0.1 wt%) occurred during this process.

Protein samples in hydrated DES were prepared by mixing the required amount of protein stock solution with water and DES to reach the desired protein concentration and water content. These samples were equilibrated for 24 hours at room temperature and stored at 4 °C prior measurement.

Aqueous protein solutions were prepared in 10 mM, pH 7, phosphate buffer following the same protocol as for the proteins in water. Both protein stock solutions and protein in buffer were stored at 4 °C between the measurements.

All samples for in house characterization were store at 4 °C and used within 72 hours to avoid any possible protein degradation.

Sample for the SANS experiments were prepared in deuterated DES and D_2O using the protocol presented for the protiated analogues. These samples were stored at -80 ˚C before measurement and used within a week of sample preparation.

All samples contained 0.2 mg/ml NaN_3 to avoid bacterial proliferation.

Methods

Freeze-drying was performed on an Epsilon 2-6D LSCplus from Martin Christ. Samples, loaded in glass vials, were loaded in the instrument tray, and quenched to -40 ˚C. Once the samples solidified at that temperature, the pressure in the instrument was gradually reduced to 0.8 mbar to sublimate the water in the sample. The temperature of the tray was kept at -15 ˚C during the primary drying stage that lasted for 24 hours. Subsequently, the temperature of the system was gradually increased to room temperature under vacuum. Finally, the instrument automatically closed the vials under inert atmosphere and the system was vented to reach ambient conditions. No bubbling was observed during any of the freeze-drying stages.

The determination of protein concentration was conducted using a ND-1000 Spectrophotometer (Saveen Werner). The absorbance was measured at 280 nm. Protein concentration was determined from the 3 measurements, and corrections for blank and solvent contribution were performed for each sample. The extinction coefficients, molecular weights and nominal concentrations are presented in Table S2.

Table S2. Parameters used to determine the concentration of each protein in solution.

Protein	ε / cm^{-1} M^{-1}	MW / g mol^{-1}	Nominal concentration / mg/ml	Nominal concentration / μM
BSA	43,824	66,400	1	15.05
Lyz	38,940	14,307	1	69.90
IgG	210,000	150,000	1	6.67

UV-vis absorption measurements were performed using the Varian Cary 50 UV-Vis and in a Jasco V770 UV-Vis/NIR spectrometer. For samples containing BSA and Lyz, Quartz cells with a 1 mm light path, 10 mm width (Hellma Analytics) were used. Samples containing IgG were measured using 0.1 mm light path Quartz cells (Hellma Analytics). The absorbance was measured in the range 190-500 nm at a scan speed of 600 nm/min, using the dual beam mode and baseline correction, and accumulated for three repeats. The data were subtracted for solvent absorption. All measurements were performed using a temperature-controlled sample stage at 25 °C. The first-derivative UV-vis spectra were calculated from the UV-vis spectra in a wavelength range between 240 and 310 nm, where most of the differences were observed. The first-derivative data were smoothed using a Gaussian function and the smoothed data were subsequently used to calculate the second-derivative UV-vis spectra. First-derivative data before and after smoothing did not show any difference in the trends nor artifacts. The position and amplitude of the tyrosine and tryptophane peaks were accurately determined by fitting the second-derivative UV-vis spectra using three Gaussian functions and a flat baseline.

Fluorescence measurements were performed using the Cary Eclipse Fluorescence Spectrometer in the 96-well plate configuration. Measurements were conducted at excitation wavelengths of either 280 nm (tryptophan, Trp, and tyrosine, Tyr) or 295 nm (Trp), with 15 scans for each measurement. Emission intensity was measured in the range 285-600 nm and 300-600 nm for each excitation wavelength, respectively, with an excitation and emission slit of 5 nm and at a scan speed of 600 nm/min. The data were normalized to the emission maximum of each spectrum and the center of spectral mass (CSM) was calculated according to the following equation:

$$CSM = \frac{\sum F_i \lambda_i}{\sum F_i}$$

where F_i is the emitted fluorescence intensity at the wavelength λ_i.

Far-UV circular dichroism (CD) measurements were performed on a Jasco J-715 spectropolarimeter at 25 ˚C. Data were collected in a wavelength range between 190 nm and 250 nm, using a scan speed of 50 nm min^{-1}, a spectral bandwidth of 1 nm, and a response time of 1 s. Each spectrum was accumulated for 3 scans. Samples were loaded in a 0.1 mm path length quartz cuvette. The signal from each corresponding buffer was subtracted from the measurements. Data are presented as mean residue ellipticity ($[\theta]_{MR}$) in deg cm^2 $dmol^{-1}$, calculated using the equation:

$$[\theta]_{MR} = \frac{\theta}{nl[BSA]}$$

where θ is the observed ellipticity in deg, n is the number of amino acid residues (583 for BSA), l is the cuvette path length, and *[BSA]* is the concentration of protein in the sample in dmol cm^{-3}.

Temperature-dependent far-UV CD data were acquired on a Jasco J-1100 spectropolarimeter equipped with a Peltier cell holder. Temperature was varied between 20 °C to 120 °C in 5 °C steps and with a heating rate of 10 °C/min. Samples were equilibrated for 1 min at each temperature before measurement. Data were collected in a wavelength range between 200 nm and 250 nm, using a scan speed of 50 nm min^{-1} and an integration time of 1 s. Spectra were accumulated for 3 scans. Samples were loaded in a quartz cuvette with a path length of 0.01 cm (0.1 mm). For the solvents with high water content, the spectra that were affected by solvent evaporation were discarded and data are available in a narrower temperature range. Data were corrected for baseline deviations and solvent contribution.

Thermodynamic parameters were determined using a two-state transition model between the protein folded state (F) and the denatured state (D).[1-3] The population of each state in the equilibrium is defined by the equilibrium constant K_D, which can be calculated using the following equation.

$$K_D = \frac{[D]}{[F]} = \frac{f_D}{1 - f_D}$$

where f_D is the fraction denatured, which is determined from the experimental intensity of the negative feature at 222 nm of the CD spectra at each temperature (θ_{222}), and that for the folded and denatured state, $\theta_{222,F}$ and $\theta_{222,D}$ respectively.

$$f_D = \frac{\theta_{222} - \theta_{222,N}}{\theta_{222,F} - \theta_{222,N}}$$

It is important to note that the folded and denatured state are defined for the boundary conditions in each solvent.

From the equilibrium constant, the free energy of denaturation ΔG_D was determined for each system using the equation:

$$\Delta G_D = -RTln(K_D)$$

The half-denaturation temperature (T_m) was evaluated as the x-axis intercept from the linear fit of ΔG_D against the temperature. The linear regression results could be used to subsequently calculate the ΔG_D for the different systems at any given temperature within the transition region.

SANS measurements were performed on D22 at the Institut Laue-Langevin (Grenoble, France). Sample were loaded in 1 mm path length, 1 cm width, quartz Hellma cells and placed in a temperature-controlled sample changer at 25 ˚C for measurement. The front and rear detectors were placed at 1.7 m and 8 m from the sample position, respectively. Measurements were conducted using a neutron wavelength of 6 Å. The raw data was reduced according to the D22 protocols using the GRASP software.[4] The reduced data is presented as scattered intensity in absolute scale, I(q) in cm^{-1}, versus momentum transfer, q in $Å^{-1}$. The configurations used provided a combined q-range of 0.006-0.65 $Å^{-1}$.

SANS data were analyzed using a combination of approaches that avoid any structural modelling assumptions. The indirect Fourier transform (IFT) is a real-space approach used to determine the pair-distance distribution function of the scatterer (*p(r)*).[5] The p(r) accounts for all distances within the scatterer and provides information on its shape and size. The function is parametrized using the maximum dimension of the scatterer (D_{max}) and a regularization constant (α). From the p(r) the radius of gyration of the scatterer (R_g) and extrapolated zero angle (forward) intensity (*I(0)*) can be calculated using the following equations:

$$R_g{}^2 = \frac{\int_0^{D_{max}} p(r) r^2 dr}{2\int_0^{D_{max}} p(r) dr}$$

$$I(0) = 4\pi \int_0^{D_{max}} p(r) dr$$

The aggregation number (N_{agg}) is a descriptive parameter of the self-association equilibrium and is calculated from I(0) using the following equation:

$$N_{agg} = \frac{V}{V_m}$$

$$V = \sqrt{\frac{I(0)\, M_w}{10^{16}\, c\, \Delta SLD^2\, N_A}}$$

where V is the experimental volume of the scatterer, V_m is the volume of the protein monomer (in cm^3), c is the concentration of protein in the sample (in g cm^{-3}), ΔSLD is the scattering excess (i.e., the difference between the scattering length density of the protein and that of the solvent, SLD_p-SLD_s, in $Å^{-2}$), and N_A is Avogadro's number. The scattering length density and monomer volume of the proteins were calculated using the sequence of each protein (PDB files: BSA – 4F5S) and the Biomolecular Scattering Length Density Calculator from the Science and Technology Facilities Council: $SLD_p=3.057\times10^{-6}$ $Å^{-2}$ and $V_m=81012.8$ $Å^3$.[6] The SLD of D_2O is 6.37×10^{-6} $Å^{-2}$.

The IFT method implemented in the GNOM program in the ATSAS 2.8.3 suite was used to calculate the pair-distance distribution functions p(r) of the scatterers.[7, 8]

The turbidimetric rate of lysis of a Micrococcus lysodeikticus suspension (Sigma-Aldrich) as catalysed by Lyz in aqueous buffer was used to determine the activity of Lyz. The absorbance of the reacting sample was recorded at 450 nm for 180 s (5 s step, 25 ˚C, Jasco V770 UV-Vis/NIR spectrometer) in a 4 mm length quartz cuvette at 25 °C. A stock solution of the cell suspension of 157 µg/ml was diluted using reaction buffer (50 mM potassium phosphate buffer, pH 7.2) to a final cell concentration of 1.27 µg/ml, giving an absorbance at 450 nm of ca. 0.6-0.7. The cell suspension was loaded in the cuvette (900 µl) and 100 µl of the Lyz sample ([Lyz]= 40 µM in each solvent) were added to the cell. Samples were quickly mixed for ca. 2 s and placed into the sample holder for measurement. Measurements of the suspended bacteria in buffer (no Lyz present) were taken as a control, showing no significant change in the absorption during the experiments. Three repeats of individual samples were performed and averaged to obtain the final values of absorbance, where the error bars represent the standard deviation of the three measurements. The activities of Lyz (U/ml) in the different systems were determined by measuring the initial rates of enzymatic lysis of the *Micrococcus lysodeikticus* upon Lyz addition. The specific activities (U/mg of protein) were obtained by normalizing the measured activity to protein concentration. Subsequently, the specific activities were normalized to the specific activity of the native Lyz (in aqueous buffer) to determine the change in the enzymatic activity.

UV-vis characterization of BSA, IgG, and Lyz in the different solvents

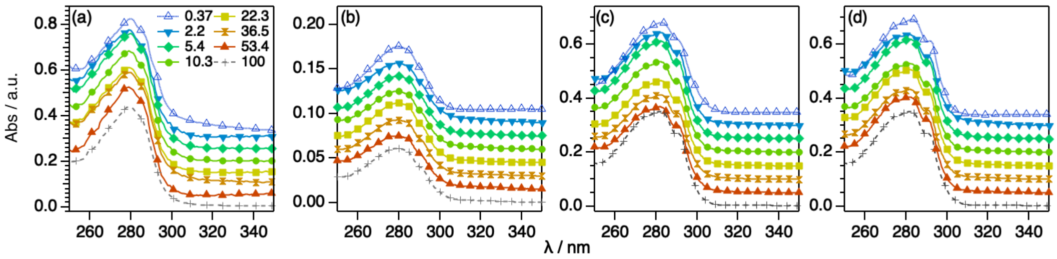

Figure S1 UV-vis spectra for (a) 100 µM BSA, (b) 20.4 µM IgG, and (c) 206 µM Lyz in 1:2:n ChCl:Glyc:H_2O; and (d) 224 µM Lyz in 1:2 ChCl:Urea:H_2O at different DES hydration expressed in wt% H_2O in the solvent. The data and results for the proteins in aqueous buffer (100 wt% H2O) are presented for comparison. Data have been offset for clarity by (a, c, d) +0.05, +0.1, +0.15, +0.20, +0.25, +0.30, and +0.35 and (b) +0.015, +0.03, +0.045, +0.06, +0.075, +0.09, and +0.105. The legend for the data is presented in panel (a).

Populations of secondary structure motifs in the different solvents

Table S3 Secondary structure content of BSA in 1:2:n ChCl:Glyc:H_2O.

wt% H_2O	α-helix / %	β-sheet / %	Turn / %	Unordered / %
0.37	49±4	10±6	12±3	29±3
2.2	41±2	20±5	12±3	27±3
5.4	17±3	35±4	9±3	39±3
10.3	30±3	29±4	10±3	31±3
22.3	40±3	21±4	11±3	28±3
36.5	62±3	8±5	11±3	19±3
53.4	55±2	11±4	12±3	22±3
100	63±3	2±3	11±3	24±2
Aq. buffer[9]	67	0	10	23

Table S4 Secondary structure content of IgG in 1:2:n ChCl:Glyc:H_2O.

wt% H_2O	α-helix / %	β-sheet / %	Turn / %	Unordered / %
0.37	1±2	72±4	13±3	14±2
2.2	2±2	69±6	9±4	20±3
5.4	0±2	41±4	4±2	55±2
10.3	0±2	43±3	6±3	51±3
22.3	0±2	58±4	12±4	30±4
36.5	0±2	68±5	14±6	18±5
53.4	0±2	73±4	16±4	11±3
100	2±2	71±4	14±4	13±3
Aq. buffer[10]	3	67	18	12

Table S5 Secondary structure content of Lyz in 1:2:n ChCl:Glyc:H_2O.

wt% H_2O	α-helix / %	β-sheet / %	Turn / %	Unordered / %
0.37	29±4	18±4	9±3	44±4
2.2	31±5	16±4	10±3	43±5
5.4	31±3	12±2	8±2	49±3
10.3	27±4	16±4	9±3	48±4
22.3	35±3	18±3	12±3	35±3
36.5	45±2	20±2	14±2	21±4
53.4	46±3	22±4	16±3	16±5
100	48±4	20±5	14±3	18±3
Aq. buffer[11]	47	27	16	10

Circular dichroism of Lyz in 1:2:n ChCl:Urea:H_2O

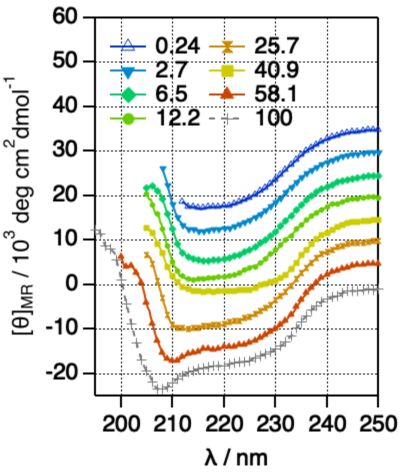

Figure S2 Mean residue ellipticity of 224 µM Lyz in 1:2:n ChCl:Urea:H_2O at different DES hydration expressed in wt% H_2O in the solvent The data for the protein in aqueous buffer (100 wt% H_2O) are presented for comparison. Data have been offset for clarity by 5×10^3, 10×10^3, 15×10^3, 20×10^3, 25×10^3, 30×10^3, and 35×10^3.

Table S6 Secondary structure content of Lyz in 1:2:n ChCl:Urea:H_2O.

wt% H_2O	α-helix / %	β-sheet / %	Turn / %	Unordered / %
40.9	26±8	24±6	8±6	42±8
58.1	41±4	30±5	10±3	19±5
100	48±4	20±4	14±3	18±4

Conformational aspects of BSA in 1:2 choline chloride:glycerol and glycerol

To corroborate the origin of the changes in protein conformation observed in the DES, we studied the effect of d-Glyc on the conformation of BSA using SANS. Data were analyzed using the IFT method as described above. Data and modes are presented in Figure S1. The results from the analysis are summarized in Table S2. These results are compared to the behavior of BSA in hydrated DES and aqueous buffer.

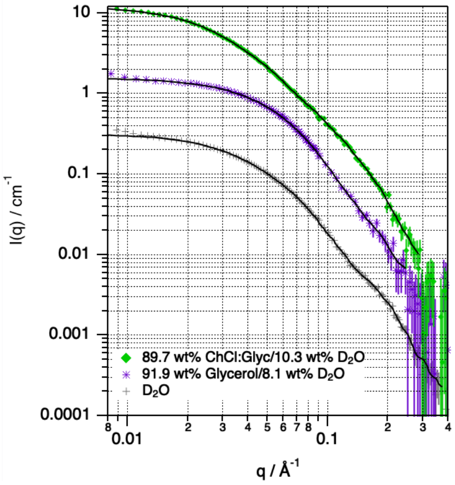

Figure S3 SANS data and models of 55 µM BSA in ChCl:Glyc:water (10.3 wt% D_2O), Glyc:water (8.1 wt% D_2O), and in buffered D_2O. The models obtained from the indirect Fourier transform (IFT) analysis are presented as solid black lines. The legend for the data is presented in the panel. Data and models have been offset for clarity by a factor of ×4, and ×16. Where not seen, error bars are within the markers.

Table S7 Parameters derived from the analysis of the SANS data presented in Figure S2.

Solvent	D_{max} / Å	r_1 / Å	N_{agg}
89.7 wt% ChCl:Glyc/10.3 wt% D_2O	193±13	45.1±2.5	1.58±0.08
91.9 wt% Glyc/8.1 wt% D_2O	126±6	33.2±2.0	1.32±0.04
Buffered D_2O	138±5	35.5±2.1	1.37±0.05

The analysis shows that the conformation of BSA in glycerol is similar to that in aqueous buffer. Only a minor compaction of the protein is observed, which has been previously observed for proteins solvated at high glycerol contents.[12] These results confirm that the main conformational changes can be mainly attributed to the ionic character of the solvent.

Thermal denaturation of BSA in neat and hydrated 1:2 choline chloride:glycerol

The thermal denaturation profiles of BSA were investigated in the different solvents using temperature-dependent far-UV circular dichroism. The spectra as a function of the wavelength are presented in Figure S2.

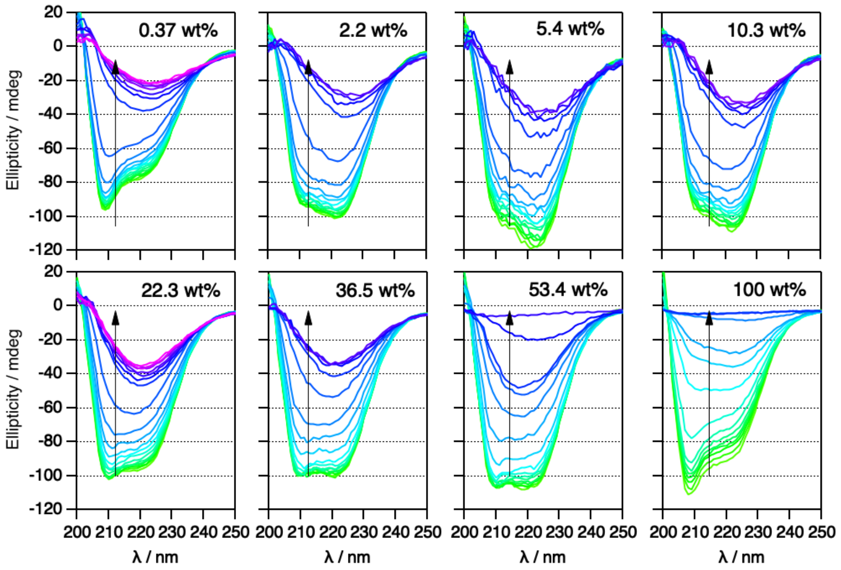

Figure S4 Temperature-dependent Far-UV CD spectra showing the evolution of 59 µM BSA in neat 1:2 choline chloride:glycerol, aqueous buffer, and 1:2:n choline chloride:glycerol:water at different hydration levels expressed in wt% H2O in the solvent. Spectra were recorded from 20 °C to 120 °C at intervals of 5 °C. The evolution in the spectra with temperature follows the direction of the arrows. For the samples with high water content, the spectra at the highest temperatures were discarded due to solvent evaporation.

The data of the free energy of denaturation (ΔG_D) against temperature as calculated from the temperature-dependent CD are shown in Figure S3. Data were fitted using liner regression.

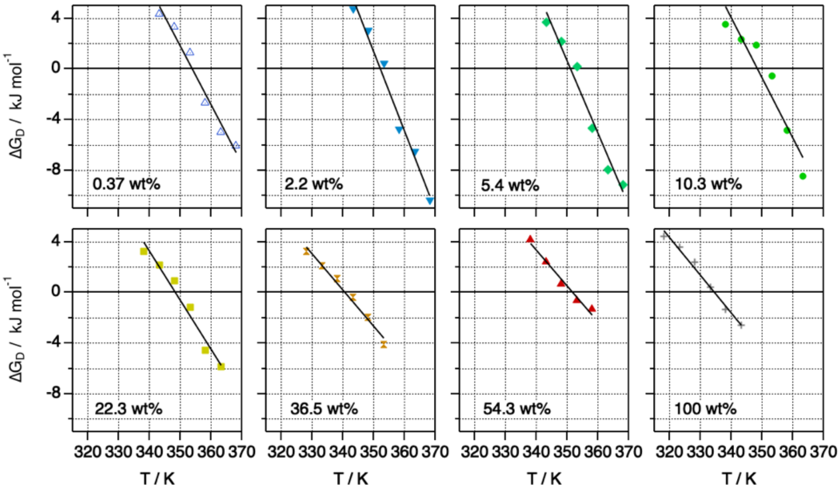

Figure S5 ΔG_D against T for BSA and fits (solid lines) at different levels of hydration, as indicated in the panels.

Secondary structure of Lyz in rehydrated DESs

Table S8 Secondary structure content of 107 µM Lyz in rehydrated DESs.

DES	wt% H_2O	α-helix / %	β-sheet / %	Turn / %	Unordered / %
1:2 ChCl:Glyc	53.4	48±6	23±6	14±5	15±4
1:2 ChCl:Urea	58.1	41±8	31±7	11±5	17±5

References

1. C. Dicko, N. Kasoju, N. Hawkins and F. Vollrath, *Soft Matter*, 2016, **12**, 255-262.
2. A. P. S. Brogan, G. Siligardi, R. Hussain, A. W. Perriman and S. Mann, *Chem. Sci.*, 2012, **3**, 1839-1846.
3. A. P. Brogan and J. P. Hallett, *J. Am. Chem. Soc.*, 2016, **138**, 4494-4501.
4. Grasp, https://www.ill.eu/users/support-labs-infrastructure/software-scientific-tools/grasp/, (accessed 2020-12-07).
5. O. Glatter, *J. Appl. Crystallogr.*, 1977, **10**, 415-421.
6. L. A. Clifton and D. Myatt, Protein Scattering Length Density Calculator v1.0, http://psldc.isis.rl.ac.uk/Psldc/, 2021).
7. M. V. Petoukhov, D. Franke, A. V. Shkumatov, G. Tria, A. G. Kikhney, M. Gajda, C. Gorba, H. D. Mertens, P. V. Konarev and D. I. Svergun, *J. Appl. Crystallogr.*, 2012, **45**, 342-350.
8. D. I. Svergun, *J. Appl. Crystallogr.*, 1992, **25**, 495-503.
9. G. Güler, M. M. Vorob'ev, V. Vogel and W. Mäntele, *Spectrochimica Acta Part A: Molecular and Biomolecular Spectroscopy*, 2016, **161**, 8-18.
10. A. Kanthe, A. Ilott, M. Krause, S. Zheng, J. Li, W. Bu, M. K. Bera, B. Lin, C. Maldarelli and R. S. Tu, *Science Advances*, 2021, **7**, eabg2873.
11. L. Sheng, J. Wang, M. Huang, Q. Xu and M. Ma, *Int. J. Biol. Macromol.*, 2016, **92**, 600-606.
12. I. Ramm, A. Sanchez-Fernandez, J. Choi, C. Lang, J. Fransson, H. Schagerlof, M. Wahlgren and L. Nilsson, *Pharmaceutics*, 2021, **13**, 1853.
